# Developmental change in look durations predicts later effortful control in toddlers at familial risk for ASD

**DOI:** 10.1186/s11689-017-9219-4

**Published:** 2018-01-29

**Authors:** Alexandra Hendry, Emily J. H. Jones, Rachael Bedford, Teodora Gliga, Tony Charman, Mark H. Johnson, the BASIS Team, S. Baron-Cohen, A. Blasi, P. Bolton, H. M. C. Cheung, K. Davies, M. Elsabbagh, J. Fernandes, I. Gammer, J. Green, J. Guiraud, S. Lloyd-Fox, M. Liew, H. Maris, L. O’Hara, G. Pasco, A. Pickles, H. Ribeiro, E. Salomone, L. Tucker, S. Wass

**Affiliations:** 1 Institute of Psychiatry, Psychology & Neuroscience, King’s College London, London, UK; 2 Centre for Brain and Cognitive Development, Department of Psychological Sciences, Birkbeck, University of London, Malet Street, London, UK

**Keywords:** Executive attention, Executive function, Effortful control, Autism, ASD, Development, Endophenotype, Infant, Sibling, Endogenous attention

## Abstract

**Background:**

Difficulties with executive functioning (EF) are common in individuals with a range of developmental disorders, including autism spectrum disorder (ASD). Interventions that target underlying mechanisms of EF early in development could be broadly beneficial, but require infant markers of such mechanisms in order to be feasible. Prospective studies of infants at high familial risk (HR) for ASD have revealed a surprising tendency for HR toddlers to show longer epochs of attention to faces than low-risk (LR) controls. In typical development, decreases in look durations towards the end of the first year of life are driven by the development of executive attention—a foundational component of EF. Here, we test the hypothesis that prolonged attention to visual stimuli (including faces) in HR toddlers reflects early differences in the development of executive attention.

**Methods:**

In a longitudinal prospective study, we used eye-tracking to record HR and LR infants’ looking behaviour to social and non-social visual stimuli at ages 9 and 15 months. At age 3 years, we assessed children with a battery of clinical research measures and collected parental report of effortful control (EC)—a temperament trait closely associated with EF and similarly contingent on executive attention.

**Results:**

Consistent with previous studies, we found an attenuated reduction in peak look durations to faces between 9 and 15 months for the HR group compared with the LR group, and lower EC amongst the HR-ASD group. In line with our hypothesis, change in peak look duration to faces between 9 and 15 months was negatively associated with EC at age 3.

**Conclusions:**

We suggest that for HR toddlers, disruption to the early development of executive attention results in an attenuated reduction in looking time to faces. Effects may be more apparent for faces due to early biases to orient towards them; further, attention difficulties may interact with earlier emerging differences in social information processing. Our finding that prolonged attention to faces may be an early indicator of disruption to the executive attention system is of potential value in screening for infants at risk for later EF difficulties and for evaluation of intervention outcomes.

**Electronic supplementary material:**

The online version of this article (10.1186/s11689-017-9219-4) contains supplementary material, which is available to authorized users.

## Background

The aetiology of autism spectrum disorder (ASD) is diverse, and the field is moving from single deficit accounts towards investigating the mechanistic underpinnings of particular behavioural and cognitive characteristics of ASD from amongst the multitude of domains affected [1]. Such accounts have potential to be useful not only in decomposing this highly heterogenous condition into clusters of characteristics that may share a genetic connection (‘endophenotypes’) [2] but also in identifying more specific mechanisms for intervention [3]. Since ASD is highly heritable [4, 5], one way of understanding early ASD is through prospective studies of infants at high familial risk for ASD (i.e. who have an older sibling with a diagnosis—henceforth ‘high-risk’ (HR) infants). Infant-sibling studies allow us to look for early atypicalities that may presage or contribute to the emergence of core symptoms and/or cognitive characteristics [6, 7].

One mechanism that has been widely implicated in ASD is executive functioning [8, 9]. Executive function (EF) is an umbrella term for the higher order cognitive functions such as working memory, inhibitory control and cognitive flexibility that are involved in planning, problem-solving and self-regulation and are conventionally measured using neuropsychological tasks in the laboratory. Effortful control (EC) is a closely related temperament trait associated with the deliberate control of behaviour and attention and is primarily measured using parent report—although behavioural measures have also been developed which show, by age of 3 years, convergence with parent report of EC and also considerable overlap with measures classically referred to as EF [10]. These top-down control mechanisms act in conjunction with bottom-up stimulus-driven processes such as visual attention to influence the way in which we filter, process and engage with the world around us—thus EFs are important to just about every aspect of life [11].

EF is highly heritable [12, 13], although specific genotype-phenotype associations have not yet been identified [14]. Difficulties with EF and EC for at least a sub-group of individuals with ASD are well-documented and emerge as early as the toddler years [15–17]. Importantly, poor EF can be considered part of the ‘broader phenotype of ASD’, in that individuals with a first-degree relative with ASD—many of whom show sub-clinical levels of the core behavioural characteristics of ASD—tend to show elevated EF problems themselves [18, 19] (although some studies have found no EF differences in ASD family members [20, 21]). Difficulties with EF are linked to poorer academic and social outcomes in typical development [11, 22] and may constitute a particular risk factor for poor life outcomes in individuals with ASD [23, 24].

Difficulties with EF may also contribute to overlap between ASD and attention deficit hyperactivity disorder (ADHD). Estimates of co-occurrence of clinical levels of ASD and ADHD symptoms range from 28 to 80% [25], and the presence of ADHD (specifically predominantly inattentive or combined sub-types) symptoms in ASD has been linked with lower EF performance [26, 27]. Evidence from studies into comorbidity of clinical diagnoses of ASD and ADHD and co-occurrence of related traits have identified poor EF as a shared endophenotype of ASD and ADHD, which may have its origins in genetically influenced difficulties with attentional control [28, 29]. In this context, EF may act as a protective factor, whereby strong EF skills better enable children to adapt in response to other perturbations to the typical developmental pathway, resulting in less-severe long-term clinical symptoms [30].

Given the impact of EF skills across domains, and their apparent early influence in a cascade of effects, interventions that target EF or foundational processes in early development could have broadly beneficial effects—since EF may be more amenable to change during this period of relative neural plasticity [31]. One significant challenge to progress has been the lack of appropriate measures for rapidly identifying the infants most at risk for poor EF (beyond the potential risk conferred by familial liability for ASD and/or ADHD, for example) [32]: due to the limitations that early social, motor and language skills place on performance, the neuropsychological and behavioural report measures of EF and EC used in later childhood are not appropriate in infancy [33]. Turning our attention to foundational processes of EF may be a more fruitful way of addressing these challenges.

Evidence from research into typical development suggests that executive attention is an important component of EF and EC [33, 34]. Executive attention is a top-down regulatory system that monitors conflict and performance feedback and that enables the endogenous control of attention by coordinating and regulating the roles of orienting and information processing. It is at least partially active by the end of the first year of life and continues to significantly develop and dominate looking behaviour throughout the second year and beyond [35–37].

Researchers have developed a range of innovative means of measuring executive attention in infancy, most of which rely on looking behaviour as an index into underlying cognitive processes without recourse to language or motor demands [37]. The simplest of these methods consider the duration of individual epochs of attention to static stimuli. Under these conditions, which minimise cues associated with exogenous attention capture (e.g. movement, luminance change, contrast change), looking is believed to be predominantly under endogenous control. In such paradigms, faces are often used as the specific stimuli because they reliably capture infants’ interest and have been found to elicit robust individual differences and predictive associations with later cognitive functioning [38] (this does mean however that some of the associations found between look duration and later cognitive functioning within habituation studies may be specific to faces).

It is widely accepted that in early infancy, looking time to stimuli is primarily constrained by information processing speed such that infants with faster processing speeds show shorter ‘peak looks’ to static stimuli (where peak look is the duration of the longest unbroken look to the stimulus) [39, 40]. Indeed, this is the logic behind the initial popularity of using habituation paradigms as early indicators of IQ. However, this association with IQ typically only holds between 2 and 8 months [41]. During the latter months of the first year of life, simple habituation-type tasks place a lower relative tax on information processing capacity and individual differences in looking behaviour become under the control of the infant. Evidence for looking behaviour becoming primarily under endogenous control at around 6–9 months comes from screen-based studies of looking behaviour [42], and behavioural studies of distractibility and focused attention [43, 44]. With this shift in the drivers of look duration, average look durations to static stimuli plateau across the last 6 months of the first year of life, but two distinct developmental trajectories can be differentiated whereby one shows a decrease in look durations from early (3–6 months) to late infancy (7–9 months) and the other an increase: the former trajectory is considered normative (characteristic of 75% of the sample) and is associated with higher scores of developmental ability than the latter [38].

Several studies have used static stimuli to test visual attention in infants at risk for ASD. Elsabbagh and colleagues used a complex visual array to measure social and non-social attention and identified longer looking to faces amongst high-risk infants [45]. In keeping with the literature on typically developing infants that suggests that longer looking in early infancy reflects information processing demands, longer looking to faces at 7 months was associated with poorer face recognition at age of 3 years [46]. Looking times further lengthened between 7 and 14 months, but 14-month looking time was not associated with poor face recognition (Gliga, personal communication)—consistent with the argument that changes in look duration in the latter half of the first year of life are less influenced by individual differences in information processing. In a clinically referred sample, toddlers with ASD showed longer habituation times to static faces at 18 to 30 months compared with typically developing controls and toddlers with developmental delays [47]. Findings such as these are seemingly contradictory to the broad evidence base for reduced social orienting and engagement evident in children with ASD aged 3 and older (e.g. [48, 49]) but are compatible with the literature outlined above that suggests longer looking in later infancy reflects atypical development of executive attention.

There is already good evidence for disruption to the development of attentional control amongst HR infants: infants who later show clinical levels of ASD symptoms tend to show difficulties with exogenously driven disengagement compared with low-risk (LR) controls, with group differences apparent at around 12–14 months [50, 51]—although one study has found group differences as early as 7 months [52]. However, there has been less emphasis on research into endogenous attentional control (i.e. spontaneous and voluntary maintenance or disengagement of attention) which may be more directly dependent on the executive attention system, compared with early emerging exogenously cued disengagement difficulties which likely rely more upon the orienting network [35].

In this study, we sought to understand whether atypical profiles of change in peak look durations across infancy were associated with disruption to the executive attention system in infants at elevated familial risk for ASD. To address this question, we chose to evaluate spontaneous looking behaviour to visual arrays comprising a mix of social (faces) and non-social (cars, phones and birds) stimuli, as the executive attention system is employed in selectively orienting to one stimulus over another when stimuli compete [53]. This task had also been previously sensitive to longer looking to faces in HR infants compared with LR infants—a finding noted at the time to be consistent with ‘an emerging overly focal attention style’ [45]. We tested whether this pattern broadly replicated in a different sample of HR and LR infants at ages 9 and 15 months (with no overlap between participants in the two studies), using peak look durations to each stimuli as the primary metric rather than overall proportion of looking time to allow us to more specifically tie our conclusions to attentional control [38, 54, 55]).

Our novel contribution was to test whether the expected relative increase in peak look durations to faces shown by HR toddlers compared with LR toddlers would be associated with lower EC at age of 3 years, and whether EC mediated any association between change in peak look duration and clinical manifestations of ASD and co-occurring ADHD symptoms (consistent with a role for EF as a broad protective factor against a range of symptomatologies [30]).

## Methods

### Participants

One hundred sixteen HR (64 male; 52 female) and 27 LR (14 male; 13 female) children took part in this longitudinal study. All HR children had at least one older sibling with a community clinical diagnosis of ASD (see Additional file 1, Participants for details). LR children were full-term infants (gestational age 38–42 weeks), had at least one older sibling and no first-degree relatives with a diagnosis of ASD and were recruited from a volunteer database at the Birkbeck Centre for Brain and Cognitive Development. At the time of enrolment, none of the infants had a known medical or developmental condition. Data for this study was collected over three visits, scheduled for when the infants reached 9 months, 15 months and 3 years of age. A sub-set (94) of the participants included in this work contributed data to a previous paper investigating risk-group differences in *fixation* durations from this task at age 9 months only [56].

### Ethics, consent and permission

This study was approved by the National Research Ethics Service. Parents provided informed written consent on behalf of themselves and their child.

### Clinical measures

At the 3-year visit, a battery of clinical research measures was used to establish ASD diagnosis: the *Autism Diagnostic Observation Schedule—Second Edition* (ADOS-2) [57], the *Autism Diagnostic Interview—Revised* (ADI-R) [58] and the *Social Communication Questionnaire* (SCQ) [59]—see Additional file 1, Clinical Assessments for details. Experienced clinical researchers (TC, GP, CC) reviewed information on ASD symptomatology (ADOS-2, ADI-R, SCQ), adaptive functioning (Vineland Adaptive Behavior Scale) [60] and developmental level (*Mullen Scales of Early Learning* (MSEL) [61] for each HR and LR child to ascertain ASD diagnostic outcome (henceforth ‘outcome group’) according to the *Diagnostic and Statistical Manual of Mental Disorders, 5th edition* (DSM-5) [62].

Five HR children did not take part in the 3-year visit but in two cases outcome group was allocated on the basis of earlier collected diagnostic information. From the 113 HR participants with outcome group classification, 17 (15 boys, 2 girls) met criteria for ASD (hereafter, HR-ASD). The remaining 96 participants (49 boys, 47 girls) did not (hereafter, HR-no ASD). Two LR children were absent in the 3-year visit but were included in outcome-group analysis as they showed typical development at the previous visits. None of the 27 LR children (14 boys, 13 girls) met DSM-5 criteria for ASD and none had a community clinical ASD diagnosis or other diagnosis for an ongoing developmental condition at the time of their 3-year visit.

### ASD and ADHD symptomatology

For comparability with parent-reported temperament (EC) and in order to minimise measurement invariance, two parent-report questionnaires were selected for analysis of continuous symptoms of ASD and ADHD symptoms at age of 3 years: The *Social Responsiveness Scale – Second Edition (SRS-2)—Preschool Form* [63] and the *Child Behavior Checklist for ages 1½ to 5* (CBCL) [64]. The SRS-2 identifies social impairment associated with ASD and quantifies its severity on sub-scales relating to social awareness, social cognition, social communication, social motivation and restricted interests and repetitive behaviour. It uses a 4-point scale from 1 (‘*not true*’) to 4 (‘*almost always true*’) across 65 items to provide a continuous measure of ASD symptom severity that extends from the clinical range to normative variation in ASD traits. The sensitivity of the SRS-2 to mild social impairment is of particular value in sibling studies where the clinical profile of symptoms in children identified with ASD at ages 2–3 years tends to be less severe than in community samples of children with an early ASD diagnosis [65].

The ADHD DSM-oriented scale of the CBCL comprises six statements that assess a child’s inattentive and hyperactive behaviour (e.g. ‘Can’t concentrate, can’t pay attention for long’, ‘Can’t sit still, restless, or hyperactive’). Parents are asked to indicate how well each statement described their child’s behaviour as observed within the past 2 months on a 3-point Likert scale. Parental assessment of hyperactive-impulsive behaviours has been demonstrated to show reasonable predictive validity during the period between 19 and 63 months [66]. Both SRS-2 and CBCL scores can be converted to age-normed *T*-scores, which were used in the analyses below.

We used the Early Learning Composite score of the MSEL to obtain a standardised measure of developmental level at every visit. Scores from the background and primary outcome characterisation measures for infants contributing experimental data are presented in Table 1.

**Table 1:** Detailed characterisation of HR subgroups and LR controls

	Low risk	High risk		
		Combined	HR-no ASD	HR-ASD
9 months				
*N*^b^ (% boys)	23 (56.52%)	94^c^ (57.45%)	75 (50.00%)	16 (88.50%)
Age in months	9.15 (0.74)	9.01 (0.81)	9.05 (0.81)	8.80 (0.83)
	*8.15–11.05*	*7.96–11.54*	*7.96–11.54*	*8.02–10.39*
MSEL ELC	112.26 (13.79)	105.93 (15.68)	107.33 (15.14)	101.81 (17.25)
	*81–133*	*65–123*	*67–123*	*65–113*
15 months				
*N*^b^ (% boys)	19 (52.63%)	97^c^ (53.61%)	80 (50.00%)	14 (85.71%)
Age in months	15.69 (.90)	15.41 (0.99)	15.46 (0.99)	15.09 (1.00)
	*14.10–17.88*	*13.87–18.94*	*13.87–18.94*	*13.97–17.36*
MSEL ELC	103.84 (16.04)^a^	93.95 (14.80)	95.96 (14.66)^a^	84.00 (12.84)
	*92–144*	*49–142*	*49–142*	*49–119*
3 years				
*N*^d^ (% boys)	24 (58%)	108 (57%)	92 (51%)	16 (88%)
Age in months	38.58 (1.38)	38.86 (2.25)	39.03 (2.16)	37.87 (2.56)
	*36–41*	*30–50*	*35–50*	*30–42*
MSEL ELC	120.21 (15.14)^a^	102.27 (24.97)	105.30 (23.22)^a^	84.81 (28.21)
	*69–141*	*49–145*	*49–145*	*49–142*
ADI-social	1.00 (1.50)^a^	3.52 (4.83)	2.02 (2.58)^a^	12.13 (5.76)
	*0–6*	*0–25*	*0–12*	*2–25*
ADI-communication	0.50 (1.06)^a^	3.96 (4.73)	2.65 (3.31)^a^	11.50 (4.69)
	*0–4*	*0–19*	*0–14*	*3–19*
ADI-RRB	0.08 (0.28)^a^	1.47 (2.42)	0.75 (1.51)^a^	5.63 (2.55)
	*0–1*	*0–10*	*0–9*	*0–10*
ADOS-Social CSS	2.58 (2.00)	2.75 (2.22)	2.51 (1.95)^a^	4.12 (3.12)
	*1–7*	*1–10*	*1–9*	*1–10*
ADOS-RRB CSS	3.58 (2.30)^a^	4.29 (2.60)	3.95 (2.59)^a^	6.25 (1.61)
	*1–7*	*1–9*	*1–9*	*5–9*
SCQ	2.71 (2.31)^a^	6.36 (6.94)	4.71 (5.35)^a^	17.93 (6.82)
	*0–9*	*0–29*	*0–27*	*0–29*

*MSEL ELC* Mullen Scales for Early Learning Early Learning Composite, *ADI* Autism Diagnostic Interview, *ADOS* Autism Diagnostic Observation Schedule, *CSS* Calibrated Severity Score, *SCQ* Social Communication Questionnaire

Standard deviations are given in parenthesis and minimum and maximum values in italics.

^a^Significant differences with the HR-ASD group at *p* < .05

^b^Sample size based on all participants that contributed look duration data at that visit, with complete measure data

^c^Includes three HR infants for whom infant data was collected but were not allocated outcome group status.

^d^Sample size based on all participants that contributed look duration data for at least one infant visit and clinical information at age 3

### Parent-reported effortful control

Parents of 3-year-olds completed the *Children’s Behavior Questionnaire – Very Short Form* (CBQ) [67]. EC scores were computed from responses to 12 questions relating to their child’s tendency (over the previous 6 months) to exercise self-restraint, concentrate intently on activities, seek out or enjoy low intensity stimulation, exhibit an awareness of subtle features or changes in the physical environment and to easily redirect attention from one activity to another. Use of parent-report measures is well-established within sibling studies (e.g. [16, 17]) and takes advantage of caregivers’ extensive opportunities to observe young children across a broad array of contexts, whilst minimising burden on the child to complete additional behavioural measures. Scores from the component scales of the CBQ EC factor have been shown to positively correlate with concurrent performance on a laboratory measure of executive attention [68] and to have high stability between the ages of 33 and 46 months [67]. Scale values were computed for participants with a minimum of 8 completed EC items, as the mean of all available items.

### Looking behaviour experimental design

#### Stimuli

**Fig. 1:**
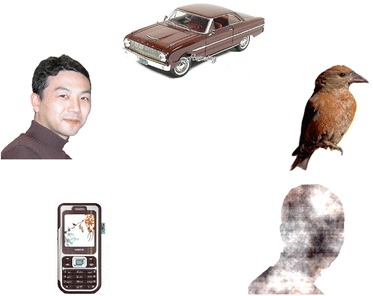
Example mixed array

The experiments were run on a Mac laptop attached to a 17-in. flat-screen monitor. The stimuli were presented in randomised order and comprised 7 ‘mixed’ and 3 ‘non-social’ arrays. Each mixed array contained 1 of 10 different faces with direct gaze (5 male, 5 female, varying ethnicity); a visual ‘noise’ image generated from the same face presented within the array by randomising the phase spectra of the face whilst keeping the amplitude and colour spectra constant to act as a control for the low-level visual properties of the face stimuli, henceforth referred to as the scrambled face stimuli; and an image of a mobile phone, a bird and a car (a different exemplar of each was used in each array), henceforth referred to as the non-social stimuli. See Fig. 1 for an example mixed array. In non-social arrays, the face image was replaced with an additional non-social stimulus. These were previously assessed for equivalent visual saliency [45], using the Saliency Toolbox [69]. The slides were counterbalanced for the location of the face within the array.

### Procedure

The task was run as part of a 20-min long battery of eye-tracking tasks, with slides evenly distributed through the battery. Before each slide was presented, a small central animation was shown, to fixate the infants’ gaze to the centre of the screen. Once gaze to the central animation was detected, the slide automatically appeared and was presented for 15 s unless the infant looked away for more than 5 s, in which case a new set of stimuli were presented. As presented in Additional file 1, Experimental data, there were no significant group differences in number of trials terminated early at either visit.

Infants were seated on their carer’s lap, 50–55 cm from the monitor, with the height adjusted to obtain good tracking of the eyes. Gaze data and pupil size were measured with either a Tobii 120 at a rate of 60 Hz (i.e. one data point every 16 ms) or a Tobii 1750 at a rate of 50 Hz (i.e. one data point every 20 ms). Look durations were calculated on the basis of the specific sample rate of the data. Additionally, main analyses were repeated with data collected with each eye-tracker type, and similar results were found in each case (see Additional file 1, Checking for an effect of eye-tracker). A 5-point calibration sequence was carried out at the start of each testing session, with recording only started when at least 4 points were marked as properly calibrated for each eye (as per [45]).

### Data processing and reduction

Look durations were calculated using an automated procedure written in MATLAB R2014b as follows: (1) Look target coordinates were calculated from an average of *x* and *y* gaze coordinates from both eyes, using single-eye coordinates where data from one eye was missing. (2) The look target was identified on the basis of rectangular areas of interest (AOIs) around each stimulus. (3) Periods of data loss (caused by blinks and or temporary loss/inaccuracy of data capture) within AOIs were linearly interpolated up to durations of 150 ms. Where gaps occurred between different AOIs, these were not interpolated. (4) Contiguous sequences to a single AOI for a minimum of 100 ms were identified as a look. The automated look duration procedure was validated using hand coding of the complete sample at the 9-month visit, as described in Additional file 1, Experimental data.

The following experimental measures were calculated and are reported in Tables 2 and 3: the total number of first looks to each AOI type as a proportion of all first looks; and peak look duration in each trial, to each AOI type (face, scrambled face and non-social). Peak look duration (i.e. duration of the longest unbroken look to a given AOI) was chosen as the primary metric as it is recommended by Colombo and colleagues as the variable that drives most of the variance in other measures of looking (such as mean look duration and total looking time) and habituation rates during infancy [38], shows robust relationships from infancy to long-term cognitive outcomes [54] and shows good test-retest reliability and consistency across different screen-based tasks amongst 11-month-olds [55]). As children participated in up to 10 trials, peak look durations were averaged across the trials to provide a more stable characterisation of individual differences. For each slide, the peak look in each category (face/scrambled face/non-social) was identified, from a minimum of two looks (> 100 ms). If no peak look was available for a particular category, the trial was excluded from the mean peak calculations for that category only. If a trial yielded no peak looks at all, it was excluded from analysis. Infants with fewer than three useable trials were excluded from analyses of peak look duration. Data meeting these criteria was obtained for 84.17% of 9-month-olds (85.19% of LR, 81.03% of HR) and 83.45% of 15-month-olds (70.37% of LR and 83.62% of HR). As presented in Additional file 1, Experimental data, there were no significant group differences in number of valid trials.

### Analytic procedure

The visual array used in this task was initially created to elicit the ‘face pop-out’ effect, a selective preference for faces in a complex visual array [70]. We therefore first evaluated whether groups differed in the proportion of first looks to the face stimuli at each time point by running generalized linear model (GLM) repeated measures ANOVAs on proportion of looks to face with time (9 months, 15 months) as a within-subject factor and group (risk/outcome) as a between-subjects factor.

We next used repeated measures ANOVAs to test for group differences in peak look duration with time (9 months, 15 months) and stimuli (faces, scrambled, non-social) as within-subject factors and group (risk/outcome) as a between-subjects factor. Characteristic of reaction time-type data, peak look durations were positively skewed—see Additional file 1, Experimental data, Table S3. Natural log transformations were used to normalise the peak look duration data before further statistical analyses were undertaken. To aid interpretation, means and standard deviations for the measured variables are presented in non-transformed form—see Table 3. Where Mauchly’s Test of Sphericity indicated that the assumption of sphericity had been violated, Greenhouse-Geisser correction was used (ε < .9). Significant group differences were followed up with post hoc Tukey tests (Levene’s test indicated that the assumption of equality of variances was not violated). During the period in which the experimental measures were captured, 54 (47%) of the high-risk families took part in a randomised controlled trial (RCT) of parent-mediated intervention [71, 72], with an additional six families enrolled in a similar non-RCT intervention [73]. Analysis was conducted to evaluate the effects of intervention (i.e. being in the treated arm of the RCT intervention or in a non-RCT intervention) on the primary experimental variables: see Additional file 1, Checking for an effect of intervention. As these factors showed no significant effects, they were removed from further analysis.

We tested for group differences in the phenotypic measures collected at age of 3 years (using Games-Howell post hoc tests for SRS-2 and CBCL-ADHD scores as Levene’s test indicated that groups showed significantly different variances). We then calculated latent change scores for peak look durations to each stimuli type—see Additional file 2 for discussion and model details—and carried out regressions of the phenotypic measures onto the computed latent change score within the structural equation model. Equivalent analysis was repeated using raw difference scores calculated by subtracting the time 1 (9-month visit) observation from the time 2 observation (15-month visit): as reported in Additional file 1, Associations between changes in looking behaviour and continuous behavioural and clinical phenotypic measures at age 3: using difference scores, consistent results were found.

SEM analyses were conducted using Mplus 7.4; all other analyses were conducted in SPSS Version 22.0.0.

## Results

### Initial orienting: the ‘face pop-out’ effect

A repeated measures ANOVA with outcome group as a between groups factor showed no main effect of outcome group (*F*(2,106) = 0.270, *p* = .764, ${\eta}_p^2$ = 0.005), no main effect of time (*F*(1,106) = 0.166, *p* = .685, ${\eta}_p^2$ = 0.002) and no interaction between time and outcome group (*F*(2106) = 0.853, *p* = .429, ${\eta}_p^2$ = 0.016) on proportion of first looks to faces—see Table 2. One sample *t* tests showed that the proportion of trials with first looks towards the face was significantly above chance level (.14) at both 9 and 15 months for all groups (LR, HR-no ASD, HR-ASD; all *p* < 0.001). This demonstrates that the face pop-out effect was observed in all groups, including those with a clinical classification of ASD by the age of 3 years.

**Table 2:** Proportion of first looks to faces, by outcome group

		Low risk	High risk		
			Combined^a^	HR-no ASD	HR-ASD
9 months	Mean (SD)	.65 (.13)	.63 (.17)	.64 (.16)	.62 (.21)
	*min–max*	.*20–.80*	.*10–1.00*	.*10–1.00*	*.10–.86*
	*N*	24	99	80	17
15 months	Mean (SD)	.63 (.12)	.66 (.14)	.66 (.14)	.65 (.15)
	*min–max*	*.29–.80*	*.00–.1.00*	*.00–.1.00*	*.43–.1.00*
	*N*	23	105	87	15

^a^Includes HR infants for whom infant data was collected but were not allocated outcome group status

**Table 3:** Peak look duration (ms) to each AOI type, by risk and outcome group

		Low risk	High risk		
			Combined^a^	HR-no ASD	HR-ASD
Face stimuli	9 months	1916.29 (949.37)	1931.74 (1238.49)	1976.76 (1266.72)	1845.04 (1161.71)
		*270–3909*	*352–7516*	*352–7516*	*395–4051*
	*N*	23	94	76	16
	15 months	1132.13 (810.70)	1771.72 (893.52)	1750.02 (878.59)	1916.50 (1082.76)
		*310–3950*	*448–5404*	*448–5404*	*760–4200*
	*N*	19	97	80	14
Scrambled face stimuli	9 months	868.35 (459.49)	758.59 (343.26)	759.18 (289.66)	794.80 (535.53)
		*260–2664*	*268–2736*	*268–1876*	*400–2736*
	*N*	22	93	74	17
	15 months	713.62 (334.68)	643.82 (289.75)	632.15 (298.15)	676.51 (229.86)
		*380–1632*	*200–2572*	*284–2572*	*200–1152*
	*N*	20	94	78	13
	Difference score	*−* 195.70 (657.01)	*−* 71.68 (352.93)	*−* 91.47 (364.37)	0.21 (309.93)
		*− 2097–860*	*− 1161–1068*	*− 1161–1068*	*− 555–517*
	*N*	16	75	60	13
Non-social stimuli	9 months	1319.12 (528.86)	1250.52 (462.82)	1229.39 (460.22)	1320.63 (509.15)
		*769–3018*	*280–2989*	*280–2989*	*651–2824*
	*N*	23	99	80	17
	15 months	1292.07 (664.91)	1367.62 (628.25)	1367.07 (675.84)	1387.40 (268.22)
		*385–2949*	*420–3987*	*420–3987*	*894–1870*
	*N*	23	105	87	15
	Difference score	*−* 91.70 (798.14)	72.27 (687.81)	87.96 (712.65)	49.76 (608.69)
		*− 1243–1573*	*− 1930–2697*	*− 1405–2697*	*− 1930–560*
	*N*	20	90	73	15

Data represent the mean duration (ms) across all trials for each participant (minimum of three trials). Standard deviations are given in parenthesis, and minimum and maximum values in italics. Difference score calculated by subtracting 9-month observation from 15-month observation

^a^Includes three HR infants for whom experimental data was collected but were not allocated outcome group status

### Peak look durations

A GLM repeated measures ANOVA on peak look duration with stimuli (faces, scrambled, non-social) and time (9 months, 15 months) as within-subject factors, and outcome group (LR, HR-no ASD, HR-ASD) as a between-subjects factor showed a main effect of time (*F*(1,76) = 6.634, *p* = .012, ${\eta}_p^2$ = 0.080) with planned simple contrasts indicating that peak look duration across outcome groups and stimuli was longer at 9 months than at 15 months—see Table 3. There was also a main effect of stimuli (Greenhouse-Geisser *F*(1.799,136.713) = 83.161, *p* < .001, ${\eta}_p^2$ = 0.522) with planned simple contrasts indicating that across outcome groups and time infants looked longer at faces than scrambled face stimuli (*p* < .001) and longer at faces than non-social stimuli (*p* = .005). There was no main effect of outcome group (*F*(2,76) = 0.149, *p* = .862, ${\eta}_p^2$ = 0.004), but there was a significant interaction effect between time and outcome group (*F*(2,76) = 4.849, *p* = .010, ${\eta}_p^2$ = 0.113). A three-way interaction of stimuli, time and outcome group was not significant (Greenhouse-Geisser *F*(3.526, 133.397) = 0.838, *p* = .491, ${\eta}_p^2$ = 0.022), nor was the two-way interaction of stimuli and outcome (Greenhouse-Geisser *F*(3.598, 136.713) = 1.509, *p =* .208 ${\eta}_p^2$ = 0.038) or stimuli and time (Greenhouse-Geisser *F*(1.763, 133.397) = 1.051, *p* = .345 ${\eta}_p^2$ = 0.014).

Post hoc ANOVAs showed that outcome groups did not differ from each other at 9 months for peak look duration to face stimuli (*F*(2,111) = 0.171, *p =* .843, ${\eta}_p^2$ = 0.003), scrambled face stimuli (*F*(2,109) = 0.646, *p* = .526, ${\eta}_p^2$ = 0.012) or non-social stimuli (*F*(1,117) = 0.964, *p* = .384, ${\eta}_p^2$ = 0.016). At 15 months, there was a significant effect of outcome group on peak look duration to faces (*F*(2,110) = 8.110, *p* = .001, ${\eta}_p^2$ = 0.129) (this significance level survives Bonferroni correction for six family-wise tests) but not on peak look durations to scrambled faces (*F*(2,108) = 0.686, *p* = .506, ${\eta}_p^2$ = 0.013) or to non-social stimuli (*F*(2,122) = 0.748, *p* = .475, ${\eta}_p^2$ = 0.012). Post hoc Tukey tests to investigate the face-specific group differences at 15 months indicated that LR toddlers made shorter peak looks to faces than both HR-no ASD toddlers (*p =* .001) and HR-ASD toddlers (*p* = .006). HR-ASD toddlers did not significantly differ from HR-no ASD toddlers (*p* = .903) with regards to peak looks to faces. Consistent with this, the interaction between group and time for peak look to faces specifically was significant at the risk group level (i.e. LR and HR) (*F*(1,93) = 4.138, *p* = .045, ${\eta}_p^2$ = 0.043) but not at the outcome group level (i.e. LR, HR-ASD, HR-no ASD) ((*F*(2, 90) = 2.374, *p* = .099, ${\eta}_p^2$ = 0.050).

**Fig. 2:**
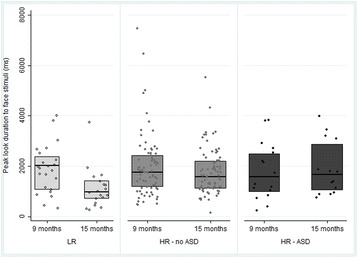
Peak look duration (ms) to face stimuli, by outcome group

In summary, compared with LR controls, the HR group (both HR-no ASD and HR-ASD) showed an altered profile of change in peak look durations to faces between 9 and 15 months, characterised by an attenuated reduction in look durations—see Table 3 and Fig. 2.

### Behavioural and clinical phenotypic measures at age of 3 years

A univariate ANOVA indicated a main effect of outcome group on EC score (*F*(2,121) = 6.304, *p* = .002, ${\eta}_p^2$ = 0.094) with post hoc Tukey tests indicating that the HR-ASD group had significantly lower EC scores than the LR group (*p* = .002) and the HR-no ASD group (*p* = .009). The HR-no ASD group did not significantly differ from the LR group (*p* = .362).

**Table 4:** Parent-reported scores of EC, ASD and ADHD at age 3, by outcome group

			High risk	
	Low risk	Combined	HR-no ASD	HR-ASD
CBQ EC score	5.15 (.65)^a^	4.82 (.79)	4.91 (.76)^a^	4.25 (.76)
	*3.75–6.33*	*2.33–6.64*	*3.25–6.64*	*2.33–5.10*
*n*	24	100	87	13
SRS total *T*-score	42.08 (4.07)^a, b^	50.21 (13.23)	46.85 (10.03)^a^	69.73 (12.13)
	*36–54*	*35–88*	*35–78*	*37–88*
*n*	24	100	85	17
CBCL-ADHD	50.96 (2.90)^a, b^	55.74 (8.01)	54.60 (6.97)^a^	63.53 (9.27)
scale *T*-score	*50–64*	50–77	*50–76*	*50–76*
*n*	24	100	88	15

Data presented for infants who contributed looking data at 9 and/or 15 months. Standard deviations are given in parenthesis, and minimum and maximum values in italics

^a^Significant differences with the HR-ASD group at *p* < .01.

^b^Significant differences with the HR-no ASD group at *p* < .01.

A univariate ANOVA indicated a main effect of outcome group on SRS *T*-score (*F*(2,121) = 44.326, *p* < .001, ${\eta}_p^2$ = 0.423) with post hoc Games-Howell tests indicating that the HR-ASD group had significantly higher SRS scores than the LR group (*p* < .001) and the HR-no ASD group (*p* < .001) — see Table 4. The HR-no ASD group also had significantly higher SRS scores than the LR group (*p* = .002). A univariate ANOVA indicated a main effect of outcome group on CBCL ADHD *T*-score (*F*(2,121) = 16.254, *p* < .001, ${\eta}_p^2$ = 0.212) with post hoc Games-Howell tests indicating that the HR-ASD group had significantly higher CBCL scores than the LR group (*p* < .001) and the HR-no ASD group (*p* < .001). The HR-no ASD group also had significantly higher CBCL scores than the LR group (*p* = .023).

### Associations between changes in looking behaviour and continuous behavioural and clinical phenotypic measures at age of 3 years

**Fig. 3:**
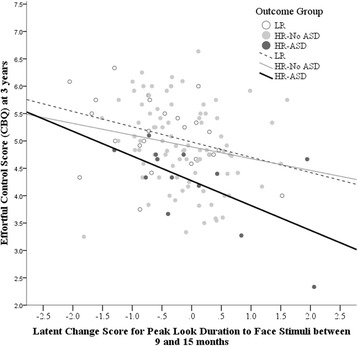
Scatter plot showing change in peak look duration to face stimuli during infancy against EC scores at age of 3 years. The lines represent the best-fit line of the model for each outcome group. A positive latent change score indicates an increase in peak look duration between 9 and 15 months and a negative score a decrease in peak look duration

Linear regression analysis was used to test the association between latent change in peak look duration (see Additional file 2) and each phenotypic measure (analysis conducted separately for each measure). Latent change in peak look duration to faces between the ages of 9 and 15 months was significantly negatively associated with EC (*β* = − .317, *R*^2^ = .10, *p* = .027) — see Fig. 3. Latent change in peak look duration to faces was not significantly associated with parent-reported ADHD symptoms (CBCL-ADHD *T*-score) (*β* = .126, *R*^2^ = .02, *p* = .476), nor with ASD symptoms (SRS *T*-score) (*β* = .159, *R*^2^ = .03, *p* = .314).

Latent change in peak look duration to non-social stimuli was not significantly associated with EC (*β* = .131, *R*^2^ = .02, *p* = .443), ADHD symptoms (*β* = − .102, *R*^2^ = .01, *p* = .516), or ASD symptoms (*β* = − .137, *R*^2^ = .02, *p* = .332). Additionally, latent change in peak look duration to scrambled face stimuli was not significantly associated with EC (*β* = − .009, *R*^2^ < .001, *p* = .958), ADHD symptoms (*β* = − .030, *R*^2^ = .001, *p* = .870), or ASD symptoms (*β* = − .080, *R*^2^ = .01, *p* = .593).

As detailed in Additional file 1, Associations between changes in looking behaviour to faces and continuous behavioural and clinical phenotypic measures at age 3: follow-up analyses, the association between latent change in peak look to faces and EC was not moderated by risk or by outcome. A similar profile of results was found using simple difference scores (see Additional file 1, Associations between changes in looking behaviour and continuous behavioural and clinical phenotypic measures at age 3: using difference scores).

## Discussion

In the current study, we asked whether the tendency for HR toddlers to look longer at faces compared with LR toddlers [45, 47] could reflect early differences in the development of executive attention. To do so, we monitored infants’ spontaneous looking behaviour to visual arrays comprising a mix of social (faces) and non-social (cars, phones and birds) stimuli at ages 9 and 15 months and collected phenotypic data on those same infants at age of 3 years. As expected based on previous work using this paradigm [45], we found a robust face pop-out effect for all risk and diagnostic groups and an interaction between risk group and time characterised by an attenuated reduction in peak look durations to faces between 9 and 15 months for the HR group (both HR-ASD and HR-no ASD) compared with the LR group. By 15 months, compared with LR infants, HR infants showed significantly longer peak look durations to faces (but no other stimulus type).

### Longer peak looks to faces relate to emerging difficulties with EF

The main aim of this study was to identify early predictors of later difficulties with EF in infants at familial risk for ASD. In particular, we focused on EC; a temperament trait associated with the deliberate control of behaviour and attention [34]. Consistent with previous studies indicating that poor EF and EC is a common feature of ASD [9], HR children diagnosed with ASD at age of 3 years showed lower EC scores compared with LR controls and the HR-no ASD group. On the basis that changes in looking behaviour during the latter part of the first year of life and into the second year are at least partly driven by increased activity of the executive attention system [35], we hypothesised that changes in peak look durations between 9 and 15 months would relate to EC scores. Our results show a significant negative association between change in peak look duration to faces between 9 and 15 months and parent-reported levels of EC, whereby lower EC at age of 3 years was associated with an attenuated decline in peak look durations over infancy. No significant associations were found between EC scores and change in peak look duration to non-social or scrambled face stimuli.

Why should the observed associations between change in peak look duration and EC be specific to faces? In the typical development literature, faces elicit robust individual differences in attentional control [38] and capture infants’ attention to a greater extent than do non-social stimuli [70], likely due to the influence of neural circuits which exert early biases to orient towards and attend to faces [74]. Indeed, in this study, and in previous work with a different sample of HR and LR infants using this paradigm [45], across risk groups and time infants looked longer at faces than to non-social stimuli, and made more first looks to faces than to any other stimulus type. Executive attention is employed in selectively orienting to one stimulus over another [53]. Thus, it can be supposed that more attentional control is required to selectively orient away from the face towards competing non-social stimuli in order to efficiently explore the array, than vice versa.

Additionally, it may be the case that social impairments may compound or amplify executive attention difficulties. Amongst older children with ASD, evidence suggests that difficulties in EF appear most pronounced for ASD groups when tasks are administered in a social context (i.e. face-to-face by a researcher), compared with computerised administration [75]. Few ASD studies to date have manipulated social and non-social conditions within a single EF task, but one such study found that on a variation of a classic EF measure, the delayed non-matching to sample task, 9-year-olds with ASD showed more difficulty in extracting a rule from social than non-social stimuli (unlike developmentally delayed controls, who showed no such effect of stimulus type) [76]. Moreover, there is some preliminary evidence that social information processing interferes with recruitment of brain regions mediating attentional control in adults with ASD [77]. Previous work has shown that amongst HR (but not LR) infants, a higher proportion of time spent looking at faces relative to other AOIs at 7 months is associated with poorer performance on a face recognition task at age 3, indicative of early-emerging face processing difficulties amongst this group [46]. Here, we have demonstrated, in a different sample, an association between change in peak look durations to faces between 8 and 15 months and EC at age of 3 years. This leads us to suggest that early difficulties in face processing interact with domain-general executive attention processes considered to drive change in look duration in late infancy.

### Relation to clinical phenotypes

We also predicted, on the basis of research suggesting that the social symptoms of ASD and attention and impulsivity problems share a phenotypic overlap with a common origin in attention difficulties [29], that a divergence from the typical trajectory of look durations between 9 and 15 months would relate to higher parent-reported ASD symptoms and attention problems at age of 3 years and that this association would become non-significant when controlling for EC. In our data, the association between change in peak look duration to faces and SRS-2 and CBCL-ADHD scores was not significant, indicating that executive attention deficits do not contribute to the core autism phenotype or to comorbid attention problems—although a null finding such as this requires replication with a larger sample and may be task and/or stimulus-specific.

Our finding that early differences in the development of endogenous attention—which we interpret as indicative of emergent executive attention difficulties—are distributed across the HR group, regardless of diagnostic outcome, is consistent with evidence that difficulties with EF are an endophenotype of ASD [18, 19]. In a large recent study, HR toddlers not showing ASD symptoms at clinical levels were found to show mean EC scores significantly lower than the LR group [15]; we found group differences in EC scores between HR-no ASD and LR 3-year-olds in the same direction as these results, but which but did not reach significance. In our sample, the HR-ASD group did show significantly lower EC scores than the HR-no ASD group, however, indicating perhaps that early executive attention difficulties distributed across the broader autism phenotype are exacerbated by some other factor for the ASD group alone, resulting in lower EC scores in that group. Given the known links between social-emotional development and EC [10], social impairments may be one such factor—and exogenous attention shifting may be another [33]. Previous work has indicated that infants who later show clinical levels of ASD symptoms have a difficulty with exogenously cued disengagement, not observed amongst HR-no ASD infants [50]. Exogenous attention shifts likely depend primarily on the earlier emerging orienting system of attention, whereas endogenous attention is contingent upon the later-emerging executive attention system (although this may in turn have its origins in the orienting system) [36]. Well-powered multivariate models are required to investigate the potential additive or interactive associations between endogenous and exogenous attention mechanisms and their association with later EC and diagnostic outcomes.

### Limitations and future directions

A strength of this study is the exploitation of a longitudinal design in order to focus on change over time as a means of investigating development [78]. However, two time points are not optimum to study developmental trajectories—particularly given that individual laboratory measures are vulnerable to moment-to-moment fluctuations in motivation and attention which increases noise in the data and likely contributed to the small effect sizes found. Therefore, future studies should consider taking measurements at additional time points during this critical period. Given the considerable resource costs and risks to attrition of adding visits to a longitudinal study, home-based testing using portable technologies may be one way to achieve this.

Previous work from our group has identified a (non-significant) trend for lower robustness (greater flicker) in data from HR relative to LR infants [56]. Given that less precise data can create the (erroneous) impression of shorter fixation durations [79], this might suggest that the evidence found here for longer looking to faces amongst HR infants compared with LR infants is, if anything, an under-estimation of group differences. Further, a minimum 4/5-point calibration standard was applied to all participants. Nevertheless, future studies should include post hoc calibration measures in order to test and control for potential group differences in recording accuracy and precision and the lack of such data in this study should be considered a limitation.

Further, as described in Additional file 1, Associations between changes in looking behaviour to faces and continuous behavioural and clinical phenotypic measures at age 3: follow-up analyses, when one extreme EC score (still within 3 SD of the HR-ASD group mean) was winsorised the direction of effect remained the same but the association now only approached significance. Therefore, replication and extension of this study is necessary to establish that findings are not sample-specific.

This study makes use of spontaneous looking to simple stimuli in order to add to the relatively sparse extant literature on the development of endogenous attention in HR infants. The use of peak look durations is well-established as a measure of endogenous attention; however, this is a simplification of a complex process: future research would benefit from the addition of other measures, such as concurrent heart rate data, to differentiate between periods of sustained attention, orienting, and disengagement occurring during individual fixations (peak or otherwise) [38]. An important next step for this area of research is to evaluate whether early differences in changes in looking behaviour to social stimuli predict atypicalities in other domains of attention later in development, and are associated with later cognitive measures of executive attention (i.e. classic EF tasks) as well as EC. Given, too, the need for caution when identifying social and attentional problems during periods of major developmental change in these domains, it would be of value to follow up this cohort with robust clinical assessments of both ASD and ADHD in early childhood.

## Conclusions

We demonstrate that HR infants as a group show an attenuated reduction in peak look duration to faces between ages 9 and 15 months (compared with LR controls) and that this attenuated reduction is associated with low EC at age 3. Informed by the literature on developmental change in looking behaviour in typical development, we conclude that these associations are indicative of early emerging differences in the development of executive attention.

We propose that the association between change in peak look duration and EC is apparent only for looks to social stimuli due to the tension between efficient exploration of the range of visual stimuli presented and the exertion of early biases to orient towards and attend to faces. Differences may be further exacerbated by an interaction between executive attention difficulties and earlier emerging differences in social information processing. This provides an explanation for the previously observed but surprising tendency for HR toddlers to make longer looks to social stimuli compared with LR controls. One avenue to consider in future studies is to co-vary for executive attention skills when considering visual attention to social stimuli in order to ascertain whether this reveals underlying differences in the expected direction (i.e. reduced orienting and attending to social stimuli).

Our line of argument has considerable practical implications for intervention in that it would suggest that infants who demonstrate longer looking to social stimuli in early toddlerhood may benefit from interventions directly targeting executive attention. Attenuated reduction in peak look duration to faces may be of use in detecting early disruption to the development of executive attention prior to the age at which classic EF and EC measures can be reliably used.

## Additional files

Additional file 1: Figure S1. Supplementary analyses. (DOCX 58 kb)

Additional file 2: Figure S2. Further explication of the Latent Change Score approach. (DOCX 51 kb)

## Abbreviations

ADHD: Attention deficit hyperactivity disorder
ADI-R: Autism Diagnostic Interview—Revised
ADOS-2: Autism Diagnostic Observation Schedule—Second edition
AOI: Area of interest
ASD: Autism spectrum disorder
CBCL: Child Behavior Checklist
CBQ: Children’s Behavior Questionnaire—Very Short Form
DSM-5: Diagnostic and Statistical Manual of Mental Disorders, 5th edition
EC: Effortful control
EF: Executive function
HR: High risk
LR: Low risk
MSEL: Mullen Scales of Early Learning
RCT: Randomised controlled trial
SCQ: Social Communication Questionnaire
SEM: Structural equation modelling
SRS-2: Social Responsiveness Scale—Second Edition
