# Supplementary material for: Developmental change in look durations predicts later effortful control in toddlers at familial risk for ASD
Source: J Neurodev Disord. 2018 Jan 29;10:3. doi: 10.1186/s11689-017-9219-4 (PMC5789678; doi:10.1186/s11689-017-9219-4)
Supplement: Supplementary file 1 — Supplementary analyses. (DOCX 58 kb) [file 11689_2017_9219_MOESM1_ESM.docx]

# Additional File 1

## Participants

A diagnosis of ASD in the older sibling (hereafter proband) was confirmed for 111 of the HR group using the Development and Wellbeing Assessment (DAWBA)[1] and/or the Social Communication Questionnaire (SCQ)[2]. Seventy-seven probands met criteria on both the DAWBA and SCQ. Eight probands scored below threshold on the SCQ but were included on the basis of meeting threshold on the DAWBA and expert opinion. For 19 probands, confirmation of local clinical diagnosis was only available for the SCQ. For 5 probands, only parent-confirmed community clinical ASD diagnosis was available. In the LR group, possible ASD in the older siblings was screened for using the SCQ, with no child scoring above the instrument cut-off for ASD (>15) (one missing). Medical history review confirmed a lack of ASD within first-degree relatives.

## Clinical Assessments

The Autism Diagnostic Observation Schedule – Second Edition (ADOS-2) [3] was used to assess current symptoms of ASD in all but 5 HR and 2 LR children. One hundred and sixteen children were administered Module 2 and 20 children were administered Module 1, on the basis of age and verbal ability. Calibrated Severity Scores for Social Affect, and Restricted and Repetitive Behaviours (RRB) were computed to provide standardised autism severity measures that account for differences in module administered, age and verbal ability [4].

The Autism Diagnostic Interview – Revised (ADI-R) [5] was completed with parents of all children. Standard algorithm scores were computed for Reciprocal Social Interaction (Social), Communication, and Restricted, Repetitive and Stereotyped Behaviours and Interests (RRB). Total scores of the SCQ were used as additional parent report measures of ASD symptoms.

These assessments were conducted without blindness to risk-group status by or under the close supervision of clinical researchers (i.e., psychologists, speech therapists) with demonstrated research-level reliability.

## Experimental data

**Validation of automated procedure using hand coded data**

The automated look duration procedure was validated using hand coding of the complete sample at the 9-month visit as follows: For each trial (i.e. presentation of a single slide), gaze direction data were run through GraFix software, which automatically parses eye-tracking data using velocity-based algorithms in order to identify possible fixations [6]. For this sample, latency was set to 60ms, velocity to 45° and displacement to 0.3°. Consecutive fixations were set to merge at a displacement level of 0.3°, variance was set to 0.4° and minimum fixation duration was set to 50ms. Automatically-detected fixations were then displayed visually, enabling the researcher to manually modify them to better fit the data and to merge fixations in close proximity into a single look. Manual modifications were conducted blind to the stimuli presented and diagnostic group.

Good inter-rater reliability was found between hand-coded and automated data: Average Measures ICC = .764 (95% CI = .662-.836) (computed on mean look durations to any AOI) using Two-Way Mixed ICC, with Consistency. A repeated measures ANOVA showed that there was no interaction between coding type and outcome group (*F*(2,113) = .094, *p*=.910 $\eta_{p}^{2}$ = .002).

*Table S1* Number of Infants Included/Excluded From Analysis, by Reason and Outcome Group

|  | **Low-Risk** | **High-Risk** | | |
| --- | --- | --- | --- | --- |
|  |  | Combined* | HR-No ASD | HR-ASD |
| **9 months** |  |  |  |  |
| Provided valid data | 23 (85.19%) | 94 (81.03%) | 76 (79.17%) | 16 (94.12%) |
| Technical problems | 0 (0.00%) | 1 (0.86%) | 1 (1.04%) | 0 (0.00%) |
| < 3 usable trials | 4 (14.81%) | 21 (18.10%) | 19 (19.79%) | 1 (5.88%) |
|  |  |  |  |  |
| **15 months** |  |  |  |  |
| Provided valid data | 19 (70.37%) | 97 (83.62%) | 80 (83.33%) | 14 (82.35%) |
| No/incomplete lab visit | 0 (0.00%) | 2 (1.72%) | 2 (2.08%) | 0 (0.00%) |
| Technical problems | 1 (3.70%) | 3 (2.59%) | 2 (2.08%) | 1 (5.88%) |
| < 3 usable trials | 7 (25.93%) | 14 (12.07%) | 12 (12.50%) | 2 (11.76%) |

Table based on number of infants providing valid peak look to face stimuli
Percentage of overall group total in brackets
*Includes 3 HR infants excluded from outcome-group analysis due to lack of clinical information

Univariate ANOVAs indicated that there were no group differences in number of valid trials at 9 months at the level of risk (*F*(1,141) = 0.002, *p* =.969 $\eta_{p}^{2}$<.001) or outcome (*F*(2,136) = 1.474, *p* =.233 $\eta_{p}^{2}$= .021), nor were there group differences in number of valid trials at 15 months at the level of risk (*F*(1,141) = 3.749, *p* =.055 $\eta_{p}^{2}$= .026) or outcome (*F*(2,136) = 2.209, *p* =.114 $\eta_{p}^{2}$= .031). We note however that the risk group difference at 15 months did approach significance. Further investigation showed that 2 of the 7 LR infants with fewer than 3 usable trials did have 1 or more valid trials, but none of these included looks to faces. Therefore it seems unlikely that the marginally increased drop-out rate for LR infants could have driven the tendency for LR infants to make shorter looks to faces compared with HR infants.

As noted in the methods section, each slide was presented for 15 seconds unless the infant looked away for more than 5 seconds, in which case a new set of stimuli were presented. Table S2 outlines how frequently this was the case.

*Table S2* Average number of trials terminated early by outcome group

|  | **Low-Risk** | **High-Risk** | | |
| --- | --- | --- | --- | --- |
|  |  | Combined | HR-No ASD | HR-ASD |
| **9 months** | .15 (.60) | .53 (1.19) | .52 (1.17) | .53 (1.01) |
| **15 months** | .22 (.64) | .37 (.94) | .30 (.86) | .71 (1.21) |

There were no significant outcome group differences in number of trials terminated early at the 9 month visit at the level of risk (*F*(1,141) = 2.676, *p*=.104 $\eta_{p}^{2}$= .019) or outcome (*F*(2,137) = 1.255 *p*=.288 $\eta_{p}^{2}$= .288), nor were there group differences in number of trials terminated early at the 15 month visit at the level of risk (*F*(1,141) = 0.609, *p*=.436 $\eta_{p}^{2}$= .004) or outcome (*F*(2,137) = 1.672, *p*=.192 $\eta_{p}^{2}$= .024).

**Distribution of experimental data**

*Table S3* Distribution statistics of peak look duration data, by stimulus type

|  | **Skewness** | | **Kurtosis** | |
| --- | --- | --- | --- | --- |
|  | Statistic | S.E | Statistic | S.E |
| **9 month visit** |  |  |  |  |
| Face | 1.665 | .224 | 4.778 | .444 |
| Scrambled face | 2.839 | .226 | 11.976 | .447 |
| Non-social | 6.406 | .218 | 56.050 | .433 |
| **15 month visit** |  |  |  |  |
| Face | 1.295 | .225 | 2.093 | .446 |
| Scrambled face | 2.956 | .226 | 15.142 | .449 |
| Non-social | 1.613 | .214 | 3.278 | .425 |

Reaction Time (RT)-type data such as looking times typically do not follow a normal (Gaussian) distribution, but rather a combination of normal and exponential distributions (Ex-Gaussian) [7]. In part this may be because such RTs are subject to influence from processes not under investigation (such as lapses of attention). To avoid data sets being unduly influenced by such processes, models can be fit to the entire distribution of response times – for example using ex-Gaussian models – rather than focusing only on participant means. However, such approaches require large numbers of trials (>100) in order to be accurate [8]. This is not feasible in infant research. An alternative is to use transformations to reduce the impact of extreme observations, or to use outlier elimination procedures to reject all values beyond a criterion number of standard deviations for a participant or condition. Marmolejo-Ramos et al. [7] have demonstrated that transformation methods are better than elimination methods in normalizing positively skewed data whilst preserving the integrity of the data. Detailed comparisons of transformations on RT data show that both inverse (reciprocal) and log transformations reduce the impact of long response times in the tails of RT distributions, leading to higher power for ANOVA analyses [9]. For this data set, natural log transformations proved sufficient to normalise the distribution of the looking time data, with the exception of one extreme value greater than 4 *SD* from the mean even after transformation (LR peak look duration to non-social stimuli at 9 months): this data point was trimmed and the remaining transformed data were used in subsequent analyses.

## Parent-reported Effortful Control

*Table S4* Number (and proportion) of infants from each outcome group with missing EC data

|  | Low-Risk |  | High-Risk | | |
| --- | --- | --- | --- | --- | --- |
|  |  | | Combined | HR-No ASD | HR-ASD |
| No items missing | 24 (89%) | | 84 (72%) | 73 (76%) | 9 (53%) |
| 1 item missing, mean scale score included | 1 (4%) | | 15 (13%) | 13 (14%) | 2 (12%) |
| 2 items missing, mean scale score included | 0 (0%) | | 5 (4%) | 3 (3%) | 2 (12%) |
| More than 3 items missing, data excluded | 0 (0%) | | 2 (2%) | 1 (1%) | 1 (6%) |
| CBQ data not available | 2 (7%) | | 10 (9%) | 6 (6%) | 3 (18%) |

As shown in Table S4, there was no statistically significant association between outcome group and missingness with regards to CBCQ-EC data (χ(8) = 13.029, p=.111).

## Replication of original face time analysis

In order to replicate the analysis of ‘face time proportion’ in an earlier cohort using this task [10], face looking time was calculated as the proportion of time spent on the face AOI relative to all target AOIs in the array (no significant effect of trial segment was found in the previous analysis therefore trial segmentation was not attempted here) – see Table S5.

*Table S5* Face time proportions

|  | **Low Risk** | **Combined*** | **No-ASD** | **ASD** |
| --- | --- | --- | --- | --- |
| **9 months**  ***N*** | .34 (.16) *.02 – .60* 23 | .36 (.18) *.01 – .71* 96 | .37 (.18) *.01 – .71* 77 | .34 (.18) *.03 – .58* 17 |
| **15 months** | .25 (.15) *.03 – .62* 23 | .36 (.15) *.00 – .72* 104 | .36 (.15) *.00 – .72* 86 | .38 (.13) *.22 – .69* 15 |

Table based on time spent on the face as a proportion of total duration of looking to all AOIs. Standard deviations are given in parenthesis, and minimum and maximum values in italics.
*Includes 3 HR infants excluded from outcome-group analysis due to lack of clinical information

A repeated measures ANOVA showed a significant main effect of risk group on proportion of face time (*F*(1,104) = 4.032, *p* = .047, $\eta_{p}^{2}$= .037) consistent with the previous finding that HR infants were more likely to sample the faces compared to other AOIs than were LR infants. The interaction of time and group did not reach significance (*F*(1,104) = 2.061, *p* = .154, $\eta_{p}^{2}$= .019) but visual examination of the data was consistent with the previously reported trend for the risk group effect in face time proportion to become more pronounced over time. – see Figure S1.


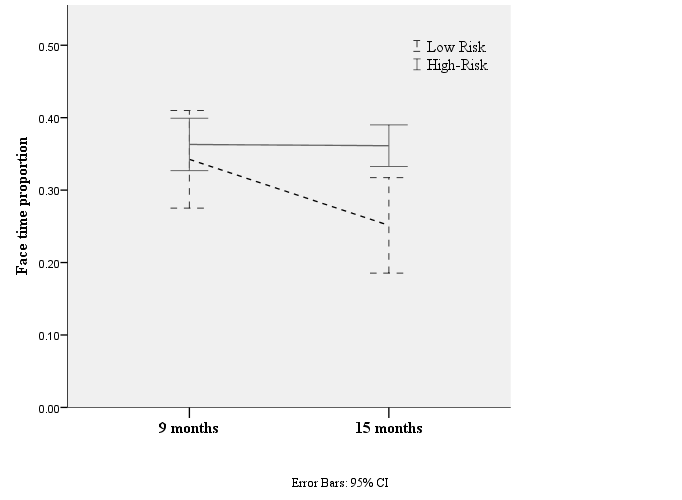


*Figure S1* Face time: proportion of time spent on face relative to other AOIs.

Consistent with previous findings, no main effect of outcome group on face time proportion was found (*F*(2,101) = 2.082, *p* = .130, $\eta_{p}^{2}$= .040) and there was no significant interaction between time and group (*F*(2,101) = 1.379, *p* = .256, $\eta_{p}^{2}$= .027)

## Risk group differences in face pop-out effect

A repeated measures ANOVA with risk group as a between groups factor indicated that there was no main effect of risk group (*F*(1,109) = 0.009, *p* = .926, $\eta_{p}^{2}$= .000), no main effect of time (*F*(1,109) < .001, *p* = .998, $\eta_{p}^{2}$< .001) and no interaction between time and risk group (*F*(1,109) = 1.767, *p* = .187, $\eta_{p}^{2}$= .016) on proportion of first looks to faces.

## Risk group differences in peak look durations

A GLM repeated measures ANOVA on peak look duration with stimuli (faces, scrambled, non-social) and time (9 months, 15 months) as within-subjects factors, and risk group (LR, HR) as a between-subjects factor showed a main effect of time (*F*(1,79) = 13.295, *p* < .001, $\eta_{p}^{2}$= .144) with planned simple contrasts indicating that peak look duration across risk groups and stimuli was longer at 9 months than at 15 months. There was a main effect of stimuli (Greenhouse-Geisser *F*(1.795,141.836) = 65.952, *p* < .001, $\eta_{p}^{2}$= .455) with pairwise comparisons indicating that across risk groups and time infants looked longer at faces than scrambled faces (*p*<.001), longer at faces than non-social stimuli (*p* =.011) and longer at non-social stimuli than scrambled faces (*p*<.001). There was no main effect of risk group (*F*(1,79) = 0.108, *p* = .743, $\eta_{p}^{2}$= .001) but there was a significant interaction effect between time and risk group (*F*(1,79) = 9.619 *p* = .003, $\eta_{p}^{2}$= .109). A three-way interaction of stimuli, time and risk group was not significant (Greenhouse-Geisser *F*(1.761, 139.093) = 1.623, *p* = .204, $\eta_{p}^{2}$= .020), nor was the two-way interaction of stimuli and risk group (Greenhouse-Geisser *F*(1.795, 141.836) = 2.496, *p =*.092 $\eta_{p}^{2}$= .031) or stimuli and time (Greenhouse-Geisser *F*(1.761, 139.093) = 1.964, *p* = .150 $\eta_{p}^{2}$= .024).

Post-hoc ANOVAs showed that at the individual time points, risk groups did not differ from each other at 9 months for peak look duration to face stimuli (*F*(1,115) = 0.018, *p =*.894, $\eta_{p}^{2}$< .001), scrambled face stimuli (*F*(1,113) = 1.554, *p* =.215, $\eta_{p}^{2}$= .014) or non-social stimuli (*F*(1,126) = 0.853, *p* =.357, $\eta_{p}^{2}$= .007). At 15 months there was a significant effect of risk group on peak look duration to faces (*F*(1,114) = 16.772, *p* <.001, $\eta_{p}^{2}$= .128) but no significant effect of risk group on peak looks to scrambled faces (*F*(1,112) = 0.824, *p* =.366 $\eta_{p}^{2}$= .007) or on peak looks to non-social stimuli (*F*(1,126) = 0.853, *p* =.357 $\eta_{p}^{2}$= .007)

## Associations between changes in looking behaviour to faces and continuous behavioural and clinical phenotypic measures at age 3: follow-up analyses

Effect of extreme values

To check whether the negative association between EC and latent change in peak look duration to faces was driven by an extreme EC score within the HR-ASD group (within 3SD of the mean for the HR-ASD group but not of the whole sample mean), the analysis was repeated with this value winsorized to the next highest value. Latent change in peak look duration to faces between the ages of 9 and 15 months remained negatively associated with EC, but the association now only approached significance (*β* = -.283, *R*^2^ = .08, *p* =.058).

Moderation effects of group on the association between latent change in peak look to faces and EC

We investigated whether the association between latent change in peak look to faces and EC was moderated by risk or by outcome, using the Hayes approach to moderation analysis [16], with syntax adapted from Stride, Gardner, Catley and Thomas [17] (model 1b, adapted for use with a latent variable). To calculate random effects with a latent variable, Analysis type = Random was used, with a Full Information Maximum Likelihood (MLF) estimator to help with model convergence. Standard errors for MLF are based on the first-order derivatives and a normal chi square statistic. The Random command does not produce standardised variables, therefore the beta values reported below are in non-standardised form.

There was no significant moderation effect of risk on the association between latent change in peak look duration to faces between the ages of 9 and 15 months and EC (β = -.237, *p* =.923), and no main effect of risk on EC (β = -.227, *p* =.337).

For the HR group there was no significant moderation effect of outcome (HR-no ASD or HR-ASD) on the association between latent change in peak look duration to faces between the ages of 9 and 15 months and EC (β = 3.008, *p* =.306), and no main effect of outcome on EC (β = .663, *p* =.120).

Regression lines for each group are shown in Figure 3.

Moderation effects of group on the association between latent change in peak look to faces and ASD symptoms

Using the same approach as described above, we investigated whether the association between latent change in peak look to faces and symptoms of ASD and ADHD at age 3 was moderated by risk or by outcome group.

There was no significant moderation effect of risk on the association between latent change in peak look duration to faces between the ages of 9 and 15 months and SRS-2 T-score (β = -2.564, *p* =.970), and no main effect of risk on SRS-2 T-score (β = 7.626, *p* =.357).

For the HR group there was no significant moderation effect of outcome on the association between latent change in peak look duration to faces between the ages of 9 and 15 months and SRS-2 T-score (β = -28.857, *p* =.592), but there was a main effect of outcome on SRS-2 T-score whereby the HR-ASD group showed higher SRS T-scores than the HR-No ASD group (β = -22.909, *p* <.001).

Moderation effects of group on the association between latent change in peak look to faces and ADHD symptoms

There was no significant moderation effect of risk on the association between latent change in peak look duration to faces between the ages of 9 and 15 months and CBCL-ADHD T-score (β = 5.778, *p* =.970), and no main effect of risk on CBCL-ADHD T-score (β = 4.910, *p* =.719).

For the HR group there was no significant moderation effect of outcome on the association between latent change in peak look duration to faces between the ages of 9 and 15 months and CBCL-ADHD T-score (β = -14.770, *p* =.470), but there was a main effect of outcome on CBCL-ADHD T-score whereby the HR-ASD group showed higher CBCL-ADHD T-scores than the HR-No ASD group (β = -8.943, *p* <.001).

## Associations between changes in looking behaviour and continuous behavioural and clinical phenotypic measures at age 3: using difference scores

Raw difference scores were calculated for peak look durations to each stimuli type by subtracting the time 1 (9 month visit) observation from the time 2 observation (15 month visit). These data were normally distributed with the exception of one outlier (> 3 *SD* below the mean) for the face stimuli (HR-No ASD group) which was excluded from analysis. Change in peak look duration between 9 and 15 months was used as the independent variable in a linear regression with the primary phenotypic measures as the dependent variable. A negative binomial model with log link function was used for the skewed count ADHD and ASD variables.

An increase in peak look duration to faces between the ages of 9 and 15 months was significantly negatively associated with EC (β = -.271, *R*^2^ = .07, *p* =.012). Increase in peak look duration to faces was not significantly associated with parent-reported ADHD symptoms (CBCL-ADHD t-score) (Wald χ^2^(1) = .041, *p* =.839), nor with ASD symptoms (SRS t-score) (Wald χ^2^(1) = .041, *p* =.733).

There were no significant associations between change in peak look duration to non-social stimuli and EC (β = .030, *R*^2^ = .001, *p* =.770), ADHD symptoms (Wald χ^2^(1) = .047, *p* =.828), or ASD symptoms (Wald χ^2^(1) = .128, *p* =.721),

Nor were there any significant associations between change in peak look duration to noise stimuli and EC (β = .111, *R*^2^ = .012, *p* =.316), ADHD symptoms (Wald χ^2^(1) = .002, *p* =.963), nor ASD symptoms (Wald χ^2^(1) = .079, *p* =.779).

## Checking for an effect of eye-tracker

Due to technical problems, approximately mid-way through the study the Tobii 1750 measuring at a rate of 50Hz was replaced with a Tobii 120 at a rate of 60Hz. We therefore checked that our main results were consistent for data collected with each eye-tracker.

For data collected at 15 months with the 60Hz eye-tracker, there remained a significant effect of risk on peak look duration to faces at 15 months (*F*(1,77) = 6.169, *p* =0.015 $\eta_{p}^{2}$= .074) whereby the LR group had shorter peak look durations (*M*=6.98 ms, *SD* = .28) than the HR group (*M*=7.44 ms, *SD* = .48). For data collected with the 50Hz eye-tracker, due to lack of power (data from only 12 LR and 25 HR 15-month-olds was collected with this eyetracker) the effect of risk on peak look duration to faces at 15 months was just beyond significance thresholds (*F*(1,35) = 4.006, *p* =0.053 $\eta_{p}^{2}$= .103) . However, the size and the direction of effect give us confidence that the results remained consistent in that the LR group had shorter peak look durations (*M*=6.77ms, *SD* = .75) than the HR group (*M*=7.16ms, *SD* = .44). Partial correlations between change score for peak look to face between 9 and 15 months and EC at age 3 showed that the association remained significant when controlling for eyetracker used at 9 and 15 months *r*(77) = -.250, *p*=.026.

## Checking for an effect of intervention

We examined the possibility that risk group differences observed at 15 months might be attributable to participation in an active intervention. An ANCOVA of peak look duration to face stimuli at 15 months by risk group with intervention participation as a covariate confirmed that the risk group differences remained significant (*F*(1,113) =13.837, *p*<.001, $\eta_{p}^{2}$= .109).

There were no group differences in change in look duration between infants who partook in an active intervention and those who did not *t*(29.196) = -1.190, p = .244.

# References: Supplementary Materials

1. Goodman R, Ford T, Richards H, Gatward R, Meltzer H. The Development and Well-Being Assessment: Description and initial validation of an integrated assessment of child and adolescent psychopathology. Journal of Child Psychology and Psychiatry and Allied Disciplines. 2000; 41:645-655.

2. Rutter M, Lord C. Social Communication Questionnaire (SCQ). Los Angeles, CA: Western Psychological Services; 2003.

3. Lord C, DiLavore PC, Risi S, Gotham K, Bishop S. Autism diagnostic observation schedule, second edition: ADOS-2. Torrance: Western Psychological Services. 2012.

4. Gotham K, Pickles A, Lord C. Standardizing ADOS Scores for a Measure of Severity in Autism Spectrum Disorders. Journal of Autism and Developmental Disorders. 2009; 39:693-705.

5. Rutter M, Le Couteur A, Lord C. Autism diagnostic interview-revised. Los Angeles, CA: Western Psychological Services; 2003. 29:30.

6. de Urabain IRS, Johnson MH, Smith TJ. GraFIX: A semiautomatic approach for parsing low- and high-quality eye-tracking data. Behavior Research Methods. 2015; 47:53-72.

7. Marmolejo-Ramos F, Cousineau D, Benites L, Maehara R. On the efficacy of procedures to normalize Ex-Gaussian distributions. Frontiers in Psychology. 2015; 5.

8. Vanselst M, Jolicoeur P. A solution to the effect of sample-size on outlier elimination. Quarterly Journal of Experimental Psychology Section a-Human Experimental Psychology. 1994; 47:631-650.

9. Ratcliff, R. Methods for dealing with reaction-time outliers. Psychological Bulletin. 1993; 114:510-532.

10. Elsabbagh M, Gliga T, Pickles A, Hudry K, Charman T, Johnson MH, BASIS Team. The development of face orienting mechanisms in infants at-risk for autism. Behavioural Brain Research. 2013; 251:147-154.

11. Karmiloff-Smith A. Development itself is the key to understanding developmental disorders. Trends in Cognitive Sciences. 1998, 2:389-398.

12. Cronbach LJ, Furby L. How we should measure" change": Or should we?. . Psychological bulletin. 1970; 74:68

13. McArdle JJ. Latent Variable Modeling of Differences and Changes with Longitudinal Data. Annual Review of Psychology. 2009; 60:577-605.

14. Hu LT, Bentler PM. Cutoff Criteria for Fit Indexes in Covariance Structure Analysis: Conventional Criteria Versus New Alternatives. Structural Equation Modeling – a Multidisciplinary Journal. 1999; 6:1-55.

15. Kievit R, Brandmaier A, Ziegler G, van Harmelen A-L, de Mooij S, Moutoussis M, Goodyer I, Bullmore E, Jones P, Fonagy P, Lindenberger U, Dolan R. Developmental cognitive neuroscience using Latent Change Score models: A tutorial and applications. bioRxiv 110429 2017 [preprint].

16. Hayes, AF. [Introduction to mediation, moderation, and conditional process
analysis: A regression-based approach](javascript:void(0)). New York, NY: Guildford Press. 2013.

17. Stride CB, Gardner SE, Catley N, & Thomas F. Mplus Code for Mediation, Moderation and Moderated Mediation Models (1 to 80). 2015. Available at: <http://www.offbeat.group.shef.ac.uk/FIO/models_and_index.pdf> [Accessed 18 August 2017].
