# Supplementary material for: Developmental change in look durations predicts later effortful control in toddlers at familial risk for ASD
Source: J Neurodev Disord. 2018 Jan 29;10:3. doi: 10.1186/s11689-017-9219-4 (PMC5789678; doi:10.1186/s11689-017-9219-4)
Supplement: Supplementary file 2 — Further explication of the Latent Change Score approach. (DOCX 51 kb) [file 11689_2017_9219_MOESM2_ESM.docx]

# Additional File 2

## Latent Change Score Model

Development is best understood through the study of change [11]. Conventional change scores have had a controversial history, with one of the most common critiques of their use being that they may increase the error in the model [12]. Latent Change Score (LCS) modelling provides a means of minimising these problems whilst retaining the value of a repeated measure by separating out the variables into structural ‘error-free’ latent components and measurement error, using the principals of Structural Equation Modelling (SEM) but requiring only a minimum of 2 time points. The latent component represents the ‘true’ change between adjacent time points [13].

In this study, we used a basic univariate latent change score model with the structure shown in figure S2. We made the following theory-based decisions regarding model structure: individual differences were estimated at time 1 but not time 2. Instead, individual differences estimated for change (mean and variance) between time 1 and 2. Change was allowed to relate to the initial level.

σ^2^ΔPLD1

1

μΔPLD1

1

β

μPLD_T2

μPLD_T1

1

σ^2^PLD_T1

σ^2^PLD_T2

*Figure S2*. Univariate Latent Change Score Model.

*Table S6* Model notations and descriptions

| **Diagram notation** | **Description** | **Mplus notation** |
| --- | --- | --- |
| **μΔPLD1** | Mean Latent Change in Peak Look Duration | [lcs*] |
| **σ^2^ΔPLD1** | Variance in Latent Change in Peak Look Duration (freely estimated in model 1) | lcs* |
| **β** | Autoregressive parameter: the extent to which change is proportional to Peak Look Duration at time 1 | Estimated by MPlus |
| **μPLD_T1** | Mean Peak Look Duration at Time 1 | [transpkfacev2] |
| **σ^2^PLD_T1** | Variance in Peak Look Duration at Time 1 | transpkfacev2 |
| **μPLD_T2** | Mean Peak Look Duration at Time 2 (15 month visit) | [transpkfacev3] |
| **σ^2^PLD_T2** | Variance in Peak Look Duration at Time 2 | transpkfacev3 |

Model syntax

lcs by transpkfacev3 @1 ;

transpkfacev3 on transpkfacev2 @1 ;

[transpkfacev3@0] ;

transpkfacev3@0 ;

[transpkfacev2*] ;

transpkfacev2* ;

lcs on transpkfacev2 @1 ;

lcs* ;

[lcs*] ;

Within the SEM, it is possible to judge the fit of a hypothesized model relative to a saturated baseline model using fit indices such as the RMSEA (root mean squared error of approximation) and the Standardized Root Mean Square Residual (SRMR). RMSEA and SRMR below .06 indicate a very good fit, whereas RMSEA below .08 and SRMR below .09 indicate a reasonable fit [14]. The RMSEA for our hypothesized model was .031, and SRMR equalled .045.

To handle incomplete data, computed maximum-likelihood estimates using the available raw data. In this procedure, each observation, or vector of observations with similar structure, is treated as a group. From these groups, the program then generates means and covariance matrices and computes a raw maximum-likelihood function. Given SEM is sensitive to departures from normality, latent change scores were computed using transformed peak look duration data. For regressions including the continuous CBQ-EC variable, which approximated a normal distribution, a Maximum Likelihood estimator was used. For regressions with skewed ordinal SRS-2 and CBCL-ADHD variables, an MLR estimator was used.

In our model, the autoregressive parameter between PeakLookDurT1 and PeakLookDurT2 was fixed to unity, thus implicitly assuming that age intervals are equidistant across individuals [15]. There were no outcome group differences in age interval (*F*(2,136) = .154, *p* = .857, $\eta_{p}^{2}$= .002), nor were there risk group differences (*F*(1,140) = .064, *p* = .800, $\eta_{p}^{2}$< .001) and overall the variance in age interval was considered low (S.D = 15.08 days). Nevertheless, 6 participants had an interval greater than 2 S.D above the mean. Analyses were re-run with these 6 participants excluded and consistent results were found: An increase in peak look duration to faces between the ages of 9 and 15 months was significantly negatively associated with EC (β = -.341, *R*^2^ = .12, *p* =.014). Latent change in peak look duration to faces was not significantly associated with parent-reported ADHD symptoms (CBCL-ADHD t-score) (β = .349, *R*^2^ = .01, *p* =.595), nor with ASD symptoms (SRS t-score) (β = .108, *R*^2^ = .01, *p* =.508).

# References: Supplementary Materials

1. Goodman R, Ford T, Richards H, Gatward R, Meltzer H. The Development and Well-Being Assessment: Description and initial validation of an integrated assessment of child and adolescent psychopathology. Journal of Child Psychology and Psychiatry and Allied Disciplines. 2000; 41:645-655.

2. Rutter M, Lord C. Social Communication Questionnaire (SCQ). Los Angeles, CA: Western Psychological Services; 2003.

3. Lord C, DiLavore PC, Risi S, Gotham K, Bishop S. Autism diagnostic observation schedule, second edition: ADOS-2. Torrance: Western Psychological Services. 2012.

4. Gotham K, Pickles A, Lord C. Standardizing ADOS Scores for a Measure of Severity in Autism Spectrum Disorders. Journal of Autism and Developmental Disorders. 2009; 39:693-705.

5. Rutter M, Le Couteur A, Lord C. Autism diagnostic interview-revised. Los Angeles, CA: Western Psychological Services; 2003. 29:30.

6. de Urabain IRS, Johnson MH, Smith TJ. GraFIX: A semiautomatic approach for parsing low- and high-quality eye-tracking data. Behavior Research Methods. 2015; 47:53-72.

7. Marmolejo-Ramos F, Cousineau D, Benites L, Maehara R. On the efficacy of procedures to normalize Ex-Gaussian distributions. Frontiers in Psychology. 2015; 5.

8. Vanselst M, Jolicoeur P. A solution to the effect of sample-size on outlier elimination. Quarterly Journal of Experimental Psychology Section a-Human Experimental Psychology. 1994; 47:631-650.

9. Ratcliff, R. Methods for dealing with reaction-time outliers. Psychological Bulletin. 1993; 114:510-532.

10. Elsabbagh M, Gliga T, Pickles A, Hudry K, Charman T, Johnson MH, BASIS Team. The development of face orienting mechanisms in infants at-risk for autism. Behavioural Brain Research. 2013; 251:147-154.

11. Karmiloff-Smith A. Development itself is the key to understanding developmental disorders. Trends in Cognitive Sciences. 1998, 2:389-398.

12. Cronbach LJ, Furby L. How we should measure" change": Or should we?. . Psychological bulletin. 1970; 74:68

13. McArdle JJ. Latent Variable Modeling of Differences and Changes with Longitudinal Data. Annual Review of Psychology. 2009; 60:577-605.

14. Hu LT, Bentler PM. Cutoff Criteria for Fit Indexes in Covariance Structure Analysis: Conventional Criteria Versus New Alternatives. Structural Equation Modeling – a Multidisciplinary Journal. 1999; 6:1-55.

15. Kievit R, Brandmaier A, Ziegler G, van Harmelen A-L, de Mooij S, Moutoussis M, Goodyer I, Bullmore E, Jones P, Fonagy P, Lindenberger U, Dolan R. Developmental cognitive neuroscience using Latent Change Score models: A tutorial and applications. bioRxiv 110429 2017 [preprint].

16. Hayes, AF. [Introduction to mediation, moderation, and conditional process
analysis: A regression-based approach](javascript:void(0)). New York, NY: Guildford Press. 2013.

17. Stride CB, Gardner SE, Catley N, & Thomas F. Mplus Code for Mediation, Moderation and Moderated Mediation Models (1 to 80). 2015. Available at: <http://www.offbeat.group.shef.ac.uk/FIO/models_and_index.pdf> [Accessed 18 August 2017].
